# The number of donor HLA-derived T cell epitopes available for indirect antigen presentation determines the risk for vascular rejection after kidney transplantation

**DOI:** 10.3389/fimmu.2022.973968

**Published:** 2022-08-30

**Authors:** Michiel G. H. Betjes, Emma T. M. Peereboom, Henny G. Otten, Eric Spierings

**Affiliations:** ^1^ Department of Internal Medicine, Section of Nephrology and Transplantation, Erasmus Medical Center (MC), University Medical Center, Rotterdam, Netherlands; ^2^ Center for Translational Immunology, University Medical Center, Utrecht, Netherlands

**Keywords:** T cell-mediated acute cellular rejection, PIRCHE-II, vascular rejection, transplantation, kidney

## Abstract

The role of the indirect T-cell recognition pathway of allorecognition in acute T cell-mediated rejection (aTCMR) is not well defined. The amount of theoretical T-cell epitopes available for indirect allorecognition can be quantified for donor-recipient combinations by the Predicted Indirectly ReCognizable HLA Epitopes algorithm (PIRCHE-II). The PIRCHE-II score was calculated for 688 donor kidney-recipient combinations and associated with the incidence of first-time diagnosed cases of TCMR. A diagnosis of TCMR was made in 182 cases; 121 cases of tubulo-interstitial rejection cases (79 cases of borderline TCMR, 42 cases of TCMR IA-B) and 61 cases of vascular TCMR (TCMR II-III). The PIRCHE-II score for donor HLA-DR/DQ (PIRCHE-II DR/DQ) was highly associated with vascular rejection. At one year after transplantation, the cumulative percentage of recipients with a vascular rejection was 12.7%, 8.6% and 2.1% within respectively the high, medium and low tertile of the PIRCHE-II DR/DQ score (p<0.001). In a multivariate regression analysis this association remained significant (p<0.001 for PIRCHE-II DR/DQ tertiles). The impact of a high PIRCHE-II DR/DQ score was mitigated by older recipient age and a living donor kidney. In conclusion, indirect antigen presentation of donor HLA-peptides may significantly contribute to the risk for acute vascular rejection.

## Introduction

Although the incidence of acute T cell-mediated rejection (aTCMR) after kidney transplantation has decreased over the last decades by more potent immune suppressive drugs, aTCMR still contribute significantly to the incidence of graft loss (1, 2). The immunological mechanism of aTCMR is characterized by cytotoxic CD8^+^ cells that recognize intact mismatched HLA class-I proteins on donor cells, a mode of recognition known as the direct pathway of allorecognition. In parallel, alloreactive CD4^+^ T cells may also play an important role in acute rejection as they either can directly invade the transplanted organ and cause rejection (3), or support the activation of the directly recognizing alloreactive cytotoxic CD8^+^ T cells. In the latter process, the indirect pathway of allorecognition is involved (4). In this pathway, mismatched donor HLA is internalized by the recipient’s antigen presenting cells (APC) and processed into peptide fragments. Such donor-HLA derived peptides can then be presented by the HLA-II molecules on the cell surface of the APC of the recipient. These activated CD4^+^ T cells subsequently support the activation and proliferation of direct alloreactive CD8^+^ T cells (5–7). At present, the relative contribution of the indirect alloreactive CD4^+^ T cells to the risk for aTCMR after kidney transplantation is largely unknown.

In order to model the indirect pathway of allorecognition by CD4^+^ T cells, an algorithm has been developed to identify the Predicted Indirectly ReCognizable HLA Epitopes that can be presented by recipient HLA class II (PIRCHE-II) (8). This algorithm allows for calculation of the T-cell epitope load score based on donor HLA peptides that can be presented by HLA class II molecules of the recipient, but are not found in the recipient’s own HLA-A, -B, -C, -DRB1 and -DQB1 alleles. The potential advantage of calculating such a score is an evaluation of antigenicity of the donor HLA molecules at greater detail, as the number of potential donor HLA-derived antigenic peptides that can be presented by the recipient HLA class II to the recipient T cells may vary substantially for a given HLA mismatch. Recent studies have shown that the PIRCHE-II score associates with *de novo* donor-specific anti-HLA antibody development and the risk for long-term graft failure after kidney transplantation (9–13). In addition, an association between the PIRCHE-II score and TCMR after liver transplantation (14, 15) and the risk for TCMR after kidney transplantation (16, 17) has been found. The latter findings are indicative of a role for indirect CD4^+^ T cell alloreactivity in TCMR as discussed by Vionet et al. (15).

In this study, we investigated whether the PIRCHE-II score, as a measure for the potential indirect CD4^+^ T cell alloreactivity, is related to the incidence of aTCMR in a large cohort of kidney transplant recipients with a complete clinical database and long-term follow-up.

## Material and methods

### Study population

In this retrospective study, we evaluated all kidney transplant recipients from the Erasmus MC between 1995 and 2005. This is a sub-cohort of the PROCARE study which included all kidney transplantations performed in the 7 transplantation centers in the Netherlands within that period (18). All transplants were performed after a negative T-cell complement dependent cytotoxicity (CDC) crossmatch. Pre-transplant sera were retrospectively assessed for the presence of HLA antibodies as previously described (18, 19). In total, 734 recipients were transplanted in our center within that period. Of all the kidney transplant donors and recipients included in this study (n=688, 46 not included because of insufficient serological or clinical data), the HLA typing was available at the serological level for a minimum of HLA-A, -B and –DR. The relevant clinical data were obtained from the Dutch Organ Transplant Registry and supplemented if needed with data from our local database, yielding a completely filled database (**Table 1**). The percentage of panel-reactive antibodies (PRA) was determined pre-transplantation and a value >4% was considered as positive. All kidney graft biopsies were performed because of clinical suspicion for rejection and no *per protocol* biopsies were done. The classification of acute T cell-medicated rejection was made using the Banff 2019 criteria. Per patient, only the data of the first kidney biopsy with a diagnosis of TCMR were used for analysis. Within the first year after transplantation, 99% of all recipients suspected of a possible rejection (decrease in eGFR or unexplained proteinuria) underwent a kidney biopsy.

**Table 1:** Clinical and demographic characteristics of 688 recipients and kidney donors.

Age recipient (average +/- SD)	45.6 years (13.6)
Age donor in years (average +/- SD)	46.6 years (14.3)
Recipient male/female ratio	48/52%
Deceased/living donor kidney	48/52%
Cold ischaemia time (average +/- SD)	10.9 hours (10.9)
Re-transplantation	18%
Mean PRA at transplantation	10%
Total HLA mismatches (mean)	2.5
HLA mismatches class I	1.7
HLA mismatches class II	0.9
Follow-up (median and IQR)	13.2 years (6–15)
Recipients with anti-HLA DSA at time transplantation	21%
Induction therapy	
- Anti-IL-2 receptor antibody	5%
Maintenance immune suppressive medication	
- steroids	90%
- tacrolimus/cyclosporine	60%/38%
- MMF/azathioprine	70%/0.5%
- other	0.9%
T cell-mediated rejection, total number	182
- borderline rejection	79 (43%)
- tubulo-interstitial rejection (TCMR1a-b)	42 (23%)
- vascular rejection (TCMR2-3)	61 (34%)

PRA:panel reactive antibodies, DSA:donor specific anti-HLA antibodies.

Patients were seen in our out-patient clinic at a regular basis at least weekly in the first month after transplantation and at least at a yearly basis beyond the first year after transplantation until death, graft loss or loss to follow-up (n=3 in the first year after transplantation and n=37 in total for lost to follow-up). The last date of follow-up was December 2021. Graft failure was defined as loss of kidney function necessitating dialysis or retransplantation. Informed consent was obtained from all participants. The clinical and research activities reported are consistent with the Principles of the Declaration of Istanbul as outlined in the ‘Declaration of Istanbul on Organ Trafficking and Transplant Tourism’ and in accordance with the declaration of Helsinki. The use of clinical data and assessment of donor-specific antibodies in stored serum samples was approved by the Research Ethics Committee for Biobanks at the Erasmus MC and the Medical Ethics Committee of the University Medical Center Utrecht.

### Identification of PIRCHE-II peptides

The PIRCHE-II peptides originating from the donor’s HLA alleles that can be presented by the recipient’s HLA class II, were identified using the PIRCHE-II algorithm version 3.3.43 (PIRCHE AG, Berlin, Germany, available *via* www.pirche.com). HLA-A, -B, -C, -DRB1, -DRB3/4/5, and -DQA1/DQB1 were taken into consideration as presented loci, and HLA-DRB1 was considered as the presenting locus. As only HLA typing data at serological level were available in our cohort, the PIRCHE-II peptides and their weights were calculated based on serological typing data, as described previously in detail (11, 20). The PIRCHE-II scores for HLA class I were calculated by adding the epitope counts originating from donor HLA-A, -B, and –C, and the PIRCHE-II scores for HLA-DR/DQ were calculated by adding the epitope counts originating from donor HLA–DR/DQ.

### Statistical analysis

Differences between groups of date were assessed by the Fisher’s exact test for categorical variables, the Mann-Whitney U test for not-normally distributed continuous variables and the Kruskall-Wallis test with post-hoc comparisons for comparing multiple groups with non-normally distributed value. All p-values were 2-tailed and the level of statistical significance was set to a p-value<0.05.

The PIRCHE-II score was used both as continuous variable and structured into tertiles for Kaplan-Meier analysis with log-rank statistics for difference between strata and as categorical variable in the Cox proportional hazards analysis. Univariate Cox proportional hazards analysis was used to identify clinical and demographic variables as given in **Table 1** for their association with TCMR-free survival for the different types of TCMR (tubulo-interstitial rejection (borderline TCMR and TCMR Banff grade IA-B) and vascular rejection (TCMR Banff grade II-III)). Variables with a p-value of 0.1 or less were considered for further analysis in a multivariate analysis to calculate hazard ratios. The type of calcineurin inhibitor used was included as strata in the model. Absence of collinearity in the model covariates was formally assessed by calculating the variance inflation factor. Statistical analysis was performed with software IBM SPSS statistics 21.

## Results

**Figure 1:**
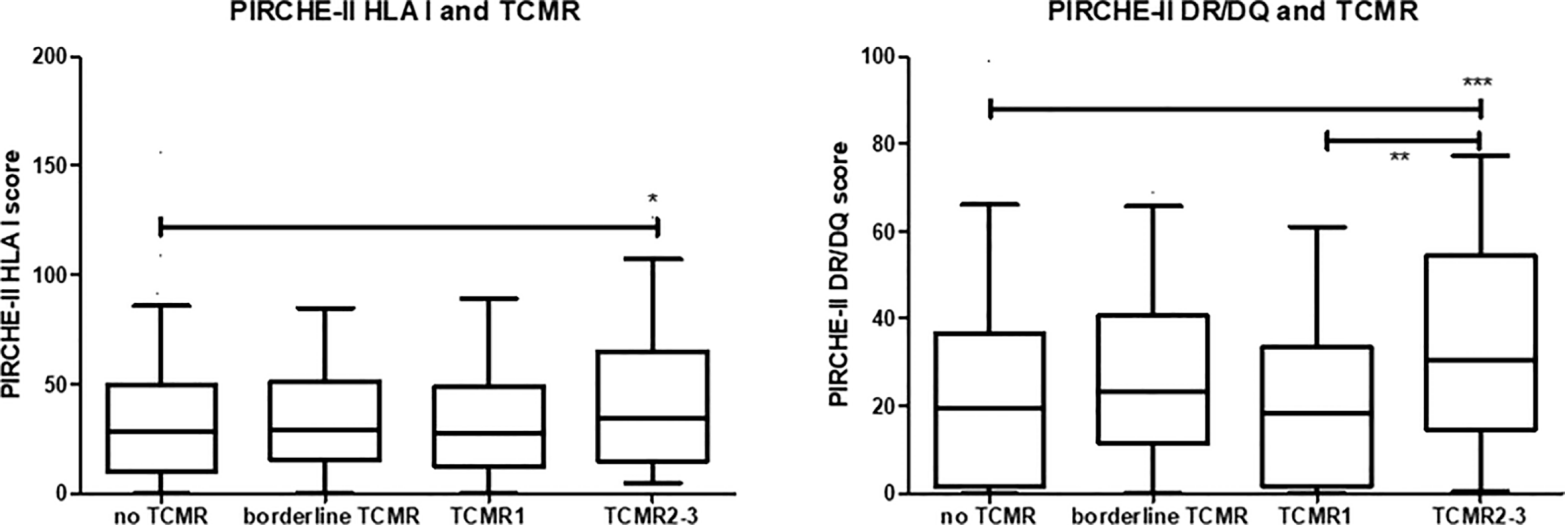
The PIRCHE-II score for donor HLA I and HLA II (DR/DQ) given in box-whisker plots for the different groups of recipients; no T cell-mediated rejection (TCMR), borderline TCMR, tubule-interstitial TCMR grade 1 and vascular TCMR grade 2-3. The p-value for the Kruskal-Waliss test for difference between multiple groups was <0.001. Post-hoc analysis for comparison between groups: *p<0.05, **p< 0.01, ***p<0.001.

In this cohort, a diagnosis of TCMR was made in 182 cases. The cohort included 121 cases of tubulo-interstitial rejection cases (79 cases of borderline rejection, 42 cases of TCMR IA-B) and 61 cases of vascular TCMR (TCMR II-III) (**Table 1** for clinical characteristics). Vascular TCMR occurred almost exclusively in the first weeks after transplantation and was rarely diagnosed thereafter. A significant difference was observed between the PIRCHE-II scores of recipients with TCMR and those with no episode of TCMR (median score 52 vs.46, p=0.01). Subsequently, the PIRCHE-II scores for the donor HLA class I derived- (loci A, B and C) and HLA class II-derived epitopes (loci DR and DQ) were separately analyzed for their relation to different types of TCMR. The PIRCHE-II HLA class I score was not significantly associated with borderline rejection, tubulo-interstitial and vascular rejection (**Figure 1**). However, the median PIRCHE-II score for HLA-DR and DQ (PIRCHE-II DR/DQ score) was significantly higher among patients with vascular rejection compared to patients with tubulo-interstitial rejection and compared to patients without rejection(median score 30 vs.18, p<0.001).

**Figure 2:**
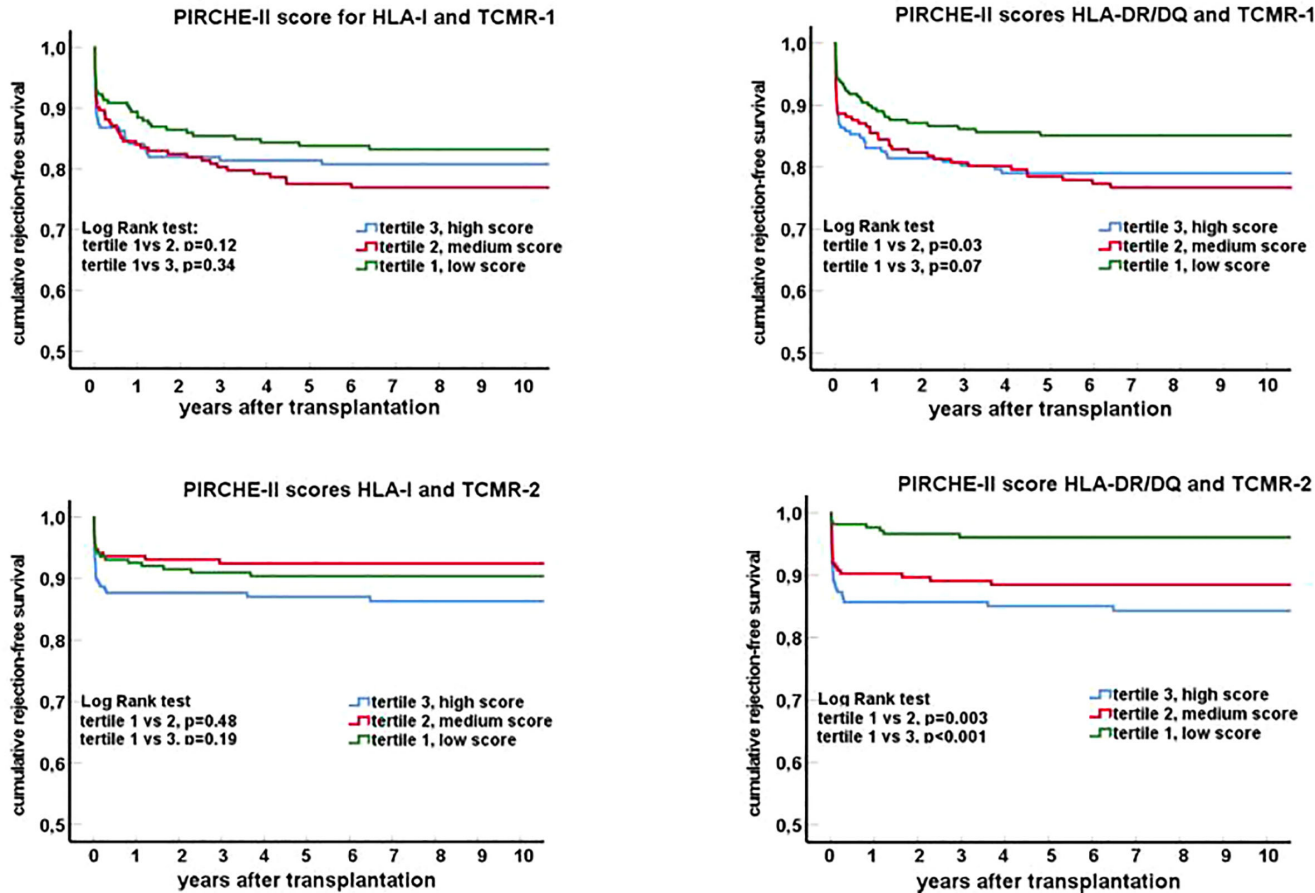
Rejection-free survival curves (Kaplan-Meier) for groups of recipients stratified for tertiles of the PIRCHE-II score for HLA I and HLA II (DR/DQ). Separate graphs show the survival curves for T cell mediated rejection: TCMR-1 in the top panels (tubulo-interstitial rejection; borderline rejection and TCMR Banff grade 1) and TCMR-2 in the lower panels (vascular rejection; TCMR Banff grade 2-3).

To assess a possible dose-response effect, the PIRCHE-II scores for HLA class I and II were divided into tertiles. Rejection-free survival curves (**Figure 2**) were made for borderline and tubulo-interstitial TCMR (TCMR Banff grade <2) and vascular rejection (TCMR grade 2-3). In particular for the PIRCHE-II DR/DQ score, a significant association was found between the tertiles and vascular rejection-free survival. One year after transplantation, the cumulative percentage of recipients with a vascular rejection was 12.7%, 8.6% and 2.1% within respectively the high, medium and low tertile of the PIRCHE-II DR/DQ score (p<0.001). In addition, a significant difference was found between the tubulo-interstitial rejection-free survival curves of patients within the lowest and the middle PIRCHE-II DR/DQ tertile (**Figure 2**, log-rank test;p=0.03). A trend for a better tubulo-interstitial rejection-free survival for the patients within the lowest PIRCHE-II DR/DQ tertile as compared to those within the highest PIRCHE-II DR/DQ tertile was observed (p=0.07).

### The PIRCHE-II DR/DQ score is an independent risk factor for vascular rejection

A uni- and multivariate logistic regression analysis was performed to calculate the hazard ratios of relevant clinical parameters for the TCMR incidence after transplantation (**Table 2** and **3**). The known risk factors for TCMR, like younger age at transplantation, deceased donor kidney vs. living donor kidney and a positive PRA (>4%) were all confirmed to have a statistically significant hazard ratio in the univariate regression analysis. In accordance with a previous publication (21), the presence of pre-transplant DSA for HLA did not affect the incidence of TCMR. An increasing PIRCHE-II DR/DQ score was strongly related to TCMR, in particular TCMR Banff grade 2-3. In a multivariate analysis, which included all univariate variables with a p-value <0.1, the hazard ratio (HR) for the highest PIRCHE-II DR/DQ tertile as compared to the lowest tertile was 1.75 for tubulo-interstitial rejection (p=0.02), and 5.34 for vascular rejection (p<0.001). Such a relation was not significantly present for the PIRCHE–II HLA I score (tubulo-interstitial rejection HR 1.10, p=0.37; vascular rejection HR 1.23, p=0.17).

**Table 2:** Uni- and multivariate Cox proportional hazard analysis for T-cell mediated rejection borderline and Banff grade 1.

	Univariate analysis		Multivariate analysis	
	Hazard ratio (95% CI)	p-value	Hazard ratio (95% CI)	p-value
Recipient age per decade	0.81 (0.71-0.93)	0.003	0.80 (0.71-0.92)	0.002
Donor age per decade	1.09 (0.95-1.26)	0.19	–	–
Deceased vs living kidney donor	1.14 (0.79-1.63)	0.47	–	–
Re-transplantation	0.93 (0.57-1.50)	0.77	–	–
PRA >4%	1.29 (0.88-1.90)	0.18	–	–
DSA pre-transplantation present	0.77 (0.48-1.24)	0.29	–	–
PIRCHE-II score HLA class-I per tertile	1.10 (0.88-1.37)	0.37	–	–
PIRCHE-II score HLA-DR/DQ per tertile		0.08		0.04
- Lowest tertile vs middle tertile	1.51 (0.95-2.40)	0.08	1.61 (1.01-2.57)	0.04
- Lowest tertile vs highest tertile	1.62 (1.03-1.54)	0.03	1.75 (1.09-2.69)	0.02

**Table 3:** Uni- and multivariate Cox proportional hazard analysis for T-cell mediated rejection Banff grade 2-3.

	Univariate analysis		Multivariate analysis	
	Hazard ratio (95% CI)	p-value	Hazard ratio (95% CI)	p-value
Recipient age per decade	0.86 (0.71-1.01)	0.10	0.81 (0.66-0.96)	0.03
Donor age per decade	1.06 (0.87-1.28)	0.54	–	–
Deceased vs living kidney donor	2.01 (1.20-3.37	0.008	2.39 (1.42-4.04)	0.002
Re-transplantation	1.25 (0.68-2.32)	0.46	–	–
PRA >4%	1.80 (1.09-3.03)	0.02	1.65 (0.99-2.77)	0.05
DSA pre-transplantation present	1.49 (0.86-2.58)	0.15	–	–
PIRCHE-II score HLA class-I per tertile	1.23 (0.98-1.67)	0.18	–	–
PIRCHE-II score HLA-DR/DQ per tertile		0.001		<0.001
- lowest tertile vs middle tertile	3.13 (1.4-7.0)	0.005	3.36 (1.49-7.58)	0.003
- lowest tertile vs highest tertile	4.46 (2.05-9.71)	<0.001	5.34 (2.44-11.7)	<0.001

**Figure 3:**
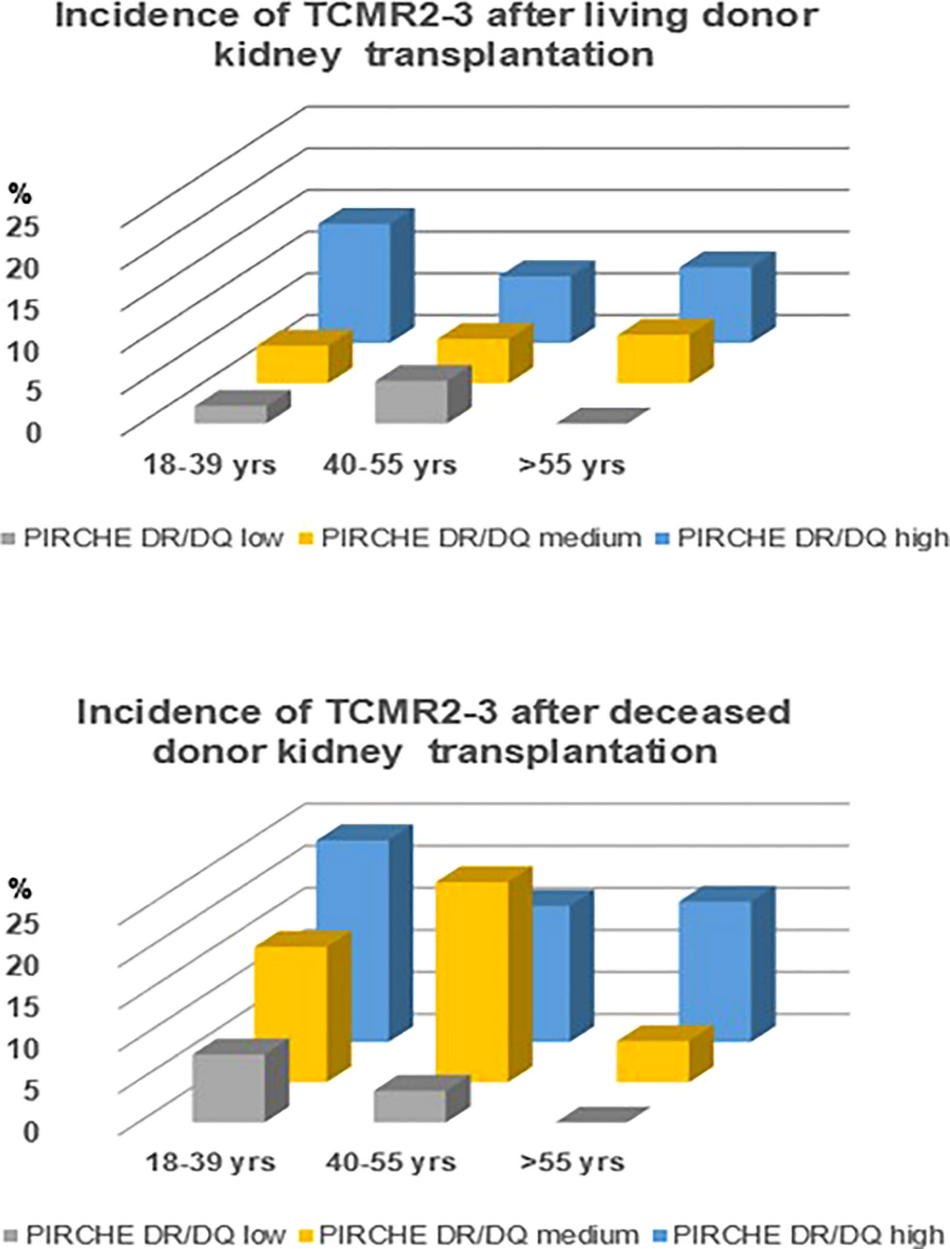
Incidence of vascular rejection (TCMR Banff grade 2-3) in relation to recipient age groups and PIRCHE-II DR/DQ tertiles for living and deceased donor kidneys.

A sensitivity analysis was done by assessing the hazard ratio per tertile of PIRCHE-II DR/DQ score in a multivariate logistic regression analysis with strata for relevant risk factors for rejection. Recipients with a cycloporine (HR 0.48, 95% CI 0.30-0.66, p=0.002) or tacrolimus-based immune suppressive regime (HR 0.44, 95% CI 0.28-0.72 p=0.002); for recipients of a living (HR 0.49, 95% CI 0.29-0.81 p=0.001) or deceased donor kidney (HR 0.50, 95% CI 0.22-0.76, p=0.018); and for different recipient age groups when divided in young (18-39 years, HR 0.46, 95% CI 0.28-0.72, p=0.005), middle age (40-55 years, HR 0.6695% CI 0.28-0.72, p=0.08) and older age (>50 years, HR 0.28, 95% CI 0.22-0.67, p=0.01). As shown in **Figure 3**, the impact of the PIRCHE-II DR/DQ score is related to the risk factors like younger age and deceased kidney donor. For instance, the risk for vascular rejection in recipients having a PIRCHE-II DR/DQ score in the highest tertile is relatively little mitigated by older age and increased by a deceased donor kidney. In contrast, no cases of vascular rejection in elderly recipients having a PIRCHE-II DR/DQ score in the lowest tertile were observed, independent of the type of donor kidney.

Finally, the effect of the PIRCHE-II score for the risk of rejection was evaluated in the fully matched recipient-donor kidney combination by serological HLA typing (n=95 transplantations). Eight cases of borderline rejection, 6 cases of TCMR Banff grade I and 2 cases of TCMR Banff grade IIA rejections were documented. These recipients had a significantly higher PIRCHE-II DR/DQ scores compared to the non-rejecting recipients (5.8 vs. 2.0, p=0.018).

## Discussion

Recently, there is a renewed interest in the long-term negative effects of TCMR. Even after T cell-depleting therapy, a definitive resolution of the rejection is sometimes not achieved and graft loss may follow in the years thereafter (1, 2, 22). Hence, a better understanding of clinical relevant HLA mismatches and mechanisms involved is still needed. In this study, we investigated whether the PIRCHE-II score, as a parameter for the potential of indirect CD4^+^ T-cell alloreactivity, is associated with aTCMR after kidney transplantation. A strong association between the PIRCHE-II score for predicted donor HLA II-derived epitopes and the incidence of TCMR was observed, in particular vascular rejection. Sensitivity analysis showed that this association was consistently found in different strata of groups of recipients based upon known risk factors for TCMR like a deceased donor kidney, a younger recipient age and cyclosporine instead of tacrolimus as calcineurin inhibitor (23, 24).

Our results are in accordance and extend previous work on the relation between the PIRCHE-II score and TCMR after liver and kidney transplantation (14–17). Vionnet *et al.* showed a significant association between PIRCHE-II and the probability score for TCMR in transplanted livers based on transcript levels of TCMR-related genes (15).

Lezoeva *et al.* showed that patients with borderline kidney rejection have a higher PIRCHE-II score compared to the entire study population, in particular in association with a high score for HLA-A (16). In contrast, Ono *et al.* observed in liver transplant recipients that the PIRCHE-II scores for HLA-DQB1 was significantly higher in the TCMR group than in the no-TCMR group, corresponding to our findings that specifically the PIRCHE-II score for the HLA-DR/DQ locus was associated with vascular rejection (14). Finally, Naef *et al.* observed an association between PIRCHE-II scores for HLA class II and TCMR as well; kidney transplant recipient with low-level BK polyomavirus-DNAemia who developed TCMR had significantly higher PIRCHE-II scores for HLA-DR compared to those who did not develop TCMR (17). A very recently published paper also investigated the relation between PIRCHE II scores and TCMR in a large cohort of kidney transplant patients (25). Similar to the results of this study, only high PIRCHE II scores for HLA class II but not class I were found to be associated with TCMR. However, whether vascular TCMR or not was specifically related to the PIRCHE II score was not stated. In addition, an unknown number of recipients were given T cell depleting induction therapy which can obscure such a relation.

**Figure 4:**
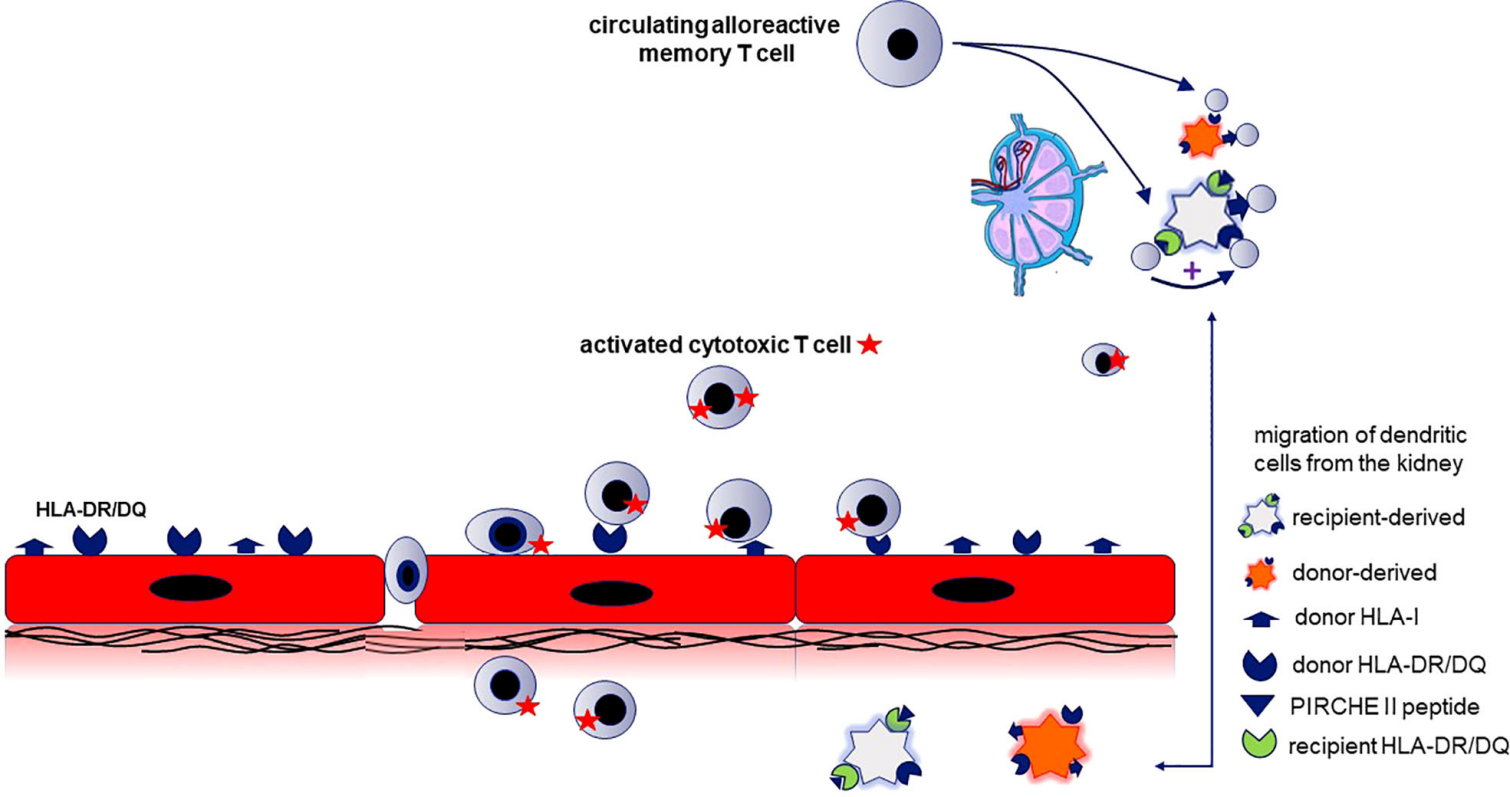
The relation between the PIRCH-II scores and acute vasculair rejection indicates that indirect antigen presentation of donor-derived MHC molecules potentiates the alloreactive cytotoxic T cell response. The mechanism proposed is semi-direct antigen presentation combined with direct antigen presentation. This requires that recipient dendritic cells (DC) cross-dressed with donor HLA-molecules (causing direct antigen presentation) is combined with indirect antigen presentation by the same DC to recipient T cells by *via* immunogenic donor HLA-II peptides (predicted by the PIRCHE score) loaded on the recipient HLA-II molecules. Renal endothelial cells (in red) constitutively express HLA-I and -II and are the source for intact donor-derived HLA molecules and the donor HLA-derived peptides that can be presented by recipient HLA-II (PIRCHE-II peptide). The recipient kidney DC (light grey cell) loaded with donor HLA II peptides on their HLA II molecules migrates to lymphoid tissue and stimulates recipient CD4^+^ T cells (indirect pathway). When the recipient dendritic cell is simultaneously cross-dressed with intact donor HLA molecules, the indirectly activated CD4^+^ T cells can provide help for directly alloreactive recipient CD4^+^ or CD8^+^ T cells (known as the semidirect pathway). The indirectly activated recipient CD4^+^ T cells may also promote the activation of alloreactive T cells recognizing donor HLA on the donor kidney-derived DC (direct pathway; orange cell). The activated alloreactive T cells attack the donor endothelial cells and migrate into the subendothelial tissue (vascular rejection).

This study emphasizes the importance of the PIRCHE-II score for HLA II donor-derived epitopes in relation to the incidence of aTCMR, especially vascular rejection, which has in general the worst prognosis of the different types of TCMR. To specifically start a vascular and not a tubulo-interstitial rejection, it seems likely that the donor endothelial cells are involved. Renal endothelial cells constitutively express HLA-II which increases shortly after transplantation and provide the source for donor HLA-II and the peptides (26, 27). Recently, Abrahimi *et al*, observed that aTCMR mediated by CD8^+^ T cells requires help from CD4^+^ T cells activated by recognition of HLA class II on the surface of donor endothelial cells (28). Following interaction with these donor HLA II molecules on the endothelial cells, the alloreactive CD4^+^ T cells become activated and can migrate across the endothelium (28, 29)(**Figure 4**). However, since the activation of these directly activated CD4^+^ T cells does not require priming by APC’s (30) it seems unlikely that the indirect pathway of allorecognition plays a role in this process. Therefore, this mode of action does not explain the association between PIRCHE-II and aTCMR.

The presentation of intact and processed MHC class I alloantigens by recipient dendritic cells (the semi-direct pathway of allorecognition (31, 32) could provide an explanation for the association between PIRCHE-II and aTCMR, since it allows linked help to be delivered by indirect-pathway CD4^+^ T cells for generating destructive cytotoxic CD8^+^ T-cell alloresponses (5, 6). An intact donor HLA molecule is acquired by the recipient APC and presented as an intact protein on the cell surface. The recipient APC loaded with donor-derived peptides on their HLA II molecules can stimulate recipient CD4^+^ T cells in e.g. the lymph node. When these APCs are simultaneously cross dressed with intact donor HLA molecules *via* the semi-direct pathway, the indirectly activated CD4^+^ T cells can provide help for directly alloreactive recipient T cells, and thereby lead to aTCMR (7). This hypothesis is illustrated in **Figure 4**.

The observed association between PIRCHE-II scores and aTCMR was significant for donor HLA DR/DQ and not for donor HLA class I. Potentially, HLA-DR/DQ could be more immunogenic than HLA class I. Although studies have shown a correlation between the degree of HLA-DR/DQ eplet molecular mismatch and T cell-mediated rejection (33, 34), it remains to be investigated whether HLA class II is more or less immunogenic than HLA class I. However, the strong association between PIRCHE-II scores and early vascular aTCMR may aid in the decision which recipients could benefit from T cell depleting induction therapy.

A limitation of this study is that we were not able to correct well for the number of HLA mismatches between donor and recipient. As the PIRCHE-II score is calculated based on HLA alleles, this score is by definition highly correlated with the number of HLA mismatches (34). Therefore, a comparative evaluation of these two tightly-associated parameters cannot be performed by standard regression analysis. One solution is to analyze the impact of the PIRCHE-II score in subcategories of the HLA-mismatch score, but this requires a database containing a sufficient large number of recipients in each subcategory. Such a cross-analysis was recently performed for the large database of the Collaborative Transplant Study and showed an independent effect of the PIRCHE-II score on graft survival, but varying in strength between different categories of HLA-mismatches (35). Our database is too small to perform reliably such an analysis but we did observe in the zero HLA mismatch stratum a significantly higher PIRCHE-II score for recipients with TCMR compared to no TCMR. In addition, kidney biopsies were only performed *for cause* and not per protocol. This could lead to an underestimation of the TCMR frequency. However, vascular rejection is in virtually all cases associated with a rapid decline in graft function and is unlikely not to be diagnosed.

In conclusion, the PIRCHE-II DR/DQ score is significantly related to TCMR, in particular acute vascular rejection. This observation indicates that an increased availability of donor HLA class II-derived epitopes for indirect antigen presentation, leading to activation of indirectly alloreactive CD4^+^ T cells, favors the development of this subtype of TCMR.

## Data availability statement

The original contributions presented in the study are included in the article/supplementary material. Further inquiries can be directed to the corresponding author.

## Ethics statement

The studies involving human participants were reviewed and approved by Research Ethics Committee for Biobanks at the Erasmus MC and the Medical Ethics Committee of the University Medical Center Utrecht. The patients/participants provided their written informed consent to participate in this study.

## Author contributions

MB: Writing, study design, data analysis. EP: Writing, data analysis. HO: Writing, data collection, data analysis. ES: Writing, data analysis. All authors contributed to the article and approved the submitted version.

## Funding

This study was supported by research funding from the Dutch Kidney Foundation Project code CP12.23 Risk assessment of kidney graft failure by HLA antibody profiling.

## Acknowledgments

The authors wish to acknowledge the contribution of the renal pathology team over the years, in particular Prof. Weening (in memoriam), I Bajema, J von der Thussen and M. Clahsen-van Groningen.

## Conflict of interest

The authors declare that the research was conducted in the absence of any commercial or financial relationships that could be construed as a potential conflict of interest.

## Publisher’s note

All claims expressed in this article are solely those of the authors and do not necessarily represent those of their affiliated organizations, or those of the publisher, the editors and the reviewers. Any product that may be evaluated in this article, or claim that may be made by its manufacturer, is not guaranteed or endorsed by the publisher.
